# Small UAV Target Detection Algorithm Using the YOLOv8n-RFL Based on Radar Detection Technology

**DOI:** 10.3390/s25165140

**Published:** 2025-08-19

**Authors:** Zhijun Shi, Zhiyong Lei

**Affiliations:** School of Mechatronic Engineering, Xi’an Technological University, Xi’an 710021, China; gemlei@xatu.edu.cn

**Keywords:** UAV, radar, YOLOv8, attention mechanism, detection

## Abstract

To improve the unmanned aerial vehicle (UAV) detection and recognition rate based on radar detection technology, this paper proposes to take the radar range-Doppler planar graph that characterizes the echo information of the UAV as the input of the improved YOLOv8 network, uses the YOLOv8n-RFL network to detect and identify the UAV target. In the detection method of the UAV target, first, we detect the echo signal of the UAV through radar, and take the received echo model as the foundation, utilize the principle of generating range-Doppler planar data to convert the received UAV echo signals into range-Doppler planar graphs, and then, use the improved YOLOv8 network to train and detect the UAV target. In the detection algorithm, the range-Doppler planar graph is taken as the input of the YOLOv8n backbone network, the UAV target is extracted from the complex background through the C2f-RVB and C2f-RVBE modules to obtain more feature maps containing multi-scale UAV feature information; the shallow features from the backbone network and deep features from the neck network are integrated through the feature semantic fusion module (FSFM) to generate high-quality fused UAV feature maps with rich details and deep semantic information, and then, the lightweight sharing detection head (LWSD) is utilized to conduct unmanned aerial vehicle (UAV) feature recognition based on the generated fused feature map. By detecting the collected echo data of the unmanned aerial vehicle (UAV), it was found that the proposed improved algorithm can effectively detect the UAV.

## 1. Introduction

With the rapid development of unmanned aerial vehicle (UAV) technology, Low–slow–small UAVs have been widely used in both military and civilian fields. In the military field, the UAVs are used for reconnaissance, surveillance, jamming and other tasks. Using unmanned aerial vehicles (UAVs) as this kind of combat mission has the advantages of low cost, high quantity and wide area cluster coverage, which poses a serious challenge to the traditional defense system. Due to the characteristics of a low-altitude flight of low, slow and small UAVs such as easy occlusion, strong concealment and small radar cross-section, there are still many deficiencies in the effective detection of UAVs at present [1,2,3]. For the detection of unmanned aerial vehicles, there are currently various detection technologies such as radar, radio, acoustics, and electro-optical. Radar detection technology is susceptible to interference from ground clutter in low-altitude complex environments. Radio detection technology cannot effectively detect silent UAVs. Acoustic detection technology is vulnerable to noise interference in cluttered environments. Photoelectric detection technology is greatly affected by weather conditions and makes it difficult to maintain stable detection performance in adverse weather [4]. The flight characteristics of low, slow and small UAV are diverse, such as low-speed flight and vertical maneuver. These characteristics make various detection devices easy to be misjudged or missed in the detection process [5]. Therefore, how to effectively detect low, slow and small UAV targets, it is a challenging conundrum.

Despite some limitations of radar detection technology, it remains the main means of obtaining unmanned aerial vehicle targets at present. Radar detects UAVs by transmitting electromagnetic waves and receiving the reflected echoes from the target. Therefore, processing and recognizing radar sensor echo signals are prerequisites for acquiring and diagnosing UAVs. Due to the inevitable external interference in the scene environment for radar detection of unmanned aerial vehicles (UAVs), how to effectively extract the echo signal of the UAV from the interference signal is a key challenge. It often requires noise reduction processing of the output signal of the radar sensor and then identification in combination with the characteristics of the UAV’s echo signal. At present, there are also many studies on radar detection and identification of unmanned aerial vehicle (UAV) targets. Yan et al. [6] analyzed the micro-Doppler signal generated by UAV rotors and designed a detection technique based on the echo Signal-to-Clutter Ratio (SCR) for relative target echo detection. Liu et al. [7] derived the frequency-domain and cepstrum expressions of echo signals based on the time-domain integral echo model for rotoring-wing UAV targets and the principle of the cepstral algorithm, analyzed the correspondence between echo signal parameters and the characteristics of the frequency-domain and cepstral, proposed a parameter estimation method for UAV echo signals, and verified the effectiveness of the method through simulation and measured data. Zhou et al. [8] proposed inputting the radar range-Doppler plane into the improved YOLOv8 model, and verified it with actual radar data, which achieved the improvement in the detection performance of small targets. Chen et al. [9] systematically reviewed and summarized the intelligent processing methods of radar weak targets from multiple aspects such as weak signal processing, image processing, and feature learning, and looked forward to future development in terms of small-sample target detection, multi-dimensional and multi-feature fusion detection, network model interpretability, and joint-driven knowledge and data. Wu et al. [10] proposed a radar modulation type recognition algorithm based on an attention-enhanced residual network to address the low recognition rate of radar signal modulation types in low signal-to-noise ratio for intelligence analysis. The algorithm utilizes the strong energy aggregation feature of smooth pseudo-Wigner–Ville distributed time-frequency transform to convert signal modulation patterns into two-dimensional time-frequency images, and integrates the convolutional attention mechanism module with a residual network to improve the effectiveness of UAV target feature extraction. Xie et al. [11], to address the problem of the low recognition rate of in-pulse modulation types of radar signals at low signal-to-noise ratio (SNR), proposed a radar signal recognition algorithm based on the time-frequency feature extraction and residual neural network. The algorithm decomposes the signal by Chirp basis through fractional Fourier transform and classifies the signal according to the different combinations of Chirp basis carrier frequency and chip frequency to improve the recognition rate of radar detection UAV targets. Liu et al. [12] proposed a method of radar signal modulation recognition based on spectral complexity for the problem that common radar signal recognition methods cannot adapt to the recognition of small pulse width signals. The method extracts the spectral complexity feature, the signal square spectral feature, the spectral peak feature, and the least squares linear fitting variance feature, and designed a tree structure flow based on spectral complexity. Wang et al. [13] proposed a multi-scale feature fusion radar target detection method based on graph neural networks for the problem of radar target detection in complex clutter environments, which uses the feature correlation between multiple pulse echoes to detect targets. These studies have achieved good results in the research of radar detection unmanned aerial vehicles (UAVs), laying a foundation for the development of new radar detection UAV targets. Especially with the development of various radar detection technologies, the current development trend is to use multi-band radar detection equipment to simultaneously obtain the echo signals of the rotor blades of unmanned aerial vehicles (UAVs) and parameters such as the motion state of UAVs, and form a detection method with multi-band radar perception. For example, Tang et al. [14] proposed a topological bidirectional fusion algorithm, this algorithm uses an improved YOLOv7-tiny to detect images of UAV, projects radar points onto images through matrix transformation, and then uses the PROSAC algorithm to fit and deflect the projected radar points to reduce the influence of hull swaying on the fusion. Zou et al. [15] researched and established the mathematical model of the quadrotor aircraft and the dual-cascade PID control system. Meanwhile, radar is used as the target detector, combined with information such as the deflection angle, the pitch angle and the distance of the target detected by radar, and a fuzzy adaptive control algorithm is introduced in the attitude control link to enhance the dynamic and steady-state performance of the unmanned aerial vehicle during flight, thereby completing the attitude and flight control of the final terminal flight guidance of the unmanned aerial vehicle (UAV).

To address the public safety challenges brought about by the extensive use of low, slow and small UAVs, achieving rapid detection and continuous tracking of low, slow and small UAV targets has become the key to intelligent prevention and control of low, slow and small targets and cost-effective countermeasures. Although deep learning technology has a powerful learning ability in the detection and recognition of unmanned aerial vehicles (UAVs), some traditional detection model networks still need to purposefully modify the existing networks according to the particularity of the detection environment to better adapt to the detection of UAVs by radar. In this paper, where we focus on detecting UAVs, the main research method and idea is to establish the echo model of the unmanned aerial vehicle (UAV). Based on the echo signal of the UAV, the echo signal is converted into the range-Doppler (RD) planar graph, and the improved YOLOv8n is used to conduct target detection on the RD graph. The highlights of this paper are as follows:

(1) We propose the YOLOv8n-RFL model to detect the UAV. The detection algorithm of UAV is based on the mathematical model of the UAV echo signal and converts the time-domain echo signal into the RD plane. Using the RD planar graph as input to the YOLOv8n backbone network, the UAV targets are extracted from the complex background by the C2f-RVB and C2f-RVBE modules, and acquire more feature maps containing multi-scale UAV feature information, improving the model’s ability of extract features.
(2) We integrate shallow features from the backbone network and the deep features from the neck network through the FSFM to generate high-quality fused feature maps with rich details and deep semantic information, and then use the LWSD detection head to perform the UAV features recognition based on the generated fused feature maps.

This paper is structured as follows: Section 2 states a mathematical model of unmanned aerial vehicle (UAV) echo based on radar detection technology; Section 3 states the UAV detection method based on YOLOv8-RFL; Section 4 states experiments and analysis. Finally, the conclusions are drawn in Section 5.

## 2. The Mathematical Model of Unmanned Aerial Vehicle (UAV) Echo Based on Radar Detection Technology

### 2.1. Basic Methods for Radar Detection of UAVs

**Figure 1:**
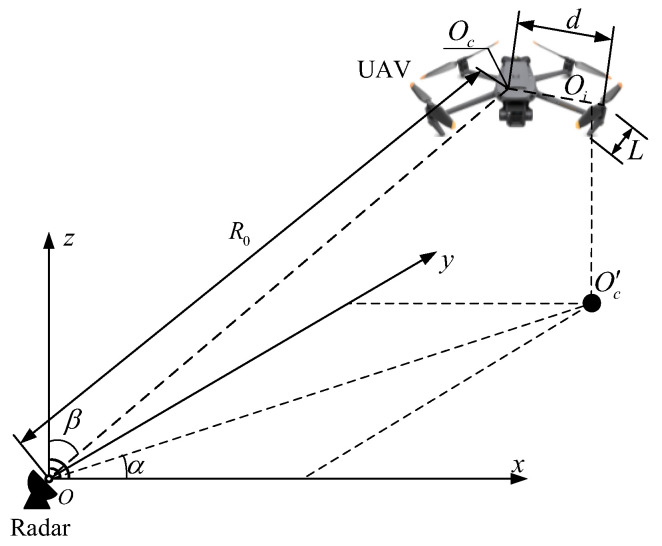
The detection principle and positional motion relationship between the UAV and radar.

The detection principle and positional motion relationship between the unmanned aerial vehicle (UAV) and the radar are shown in Figure 1. *oxyz* is the coordinate system for the radar deployment position, the radar is located at the origin of coordinate system O, and the coordinates of the center of the UAV are $O_{c}$. The coordinates of the *i*-th rotation axis center of the UAV are $O_{i}$. The azimuth angle between the radar and the UAV is α, and the pitch angle is β. The straight-line distance between the radar and the center of the UAV is $R_{0}$, and ${O^{\prime }}_{c}$ is the projection point of the unmanned aerial vehicle center on the *xoy* plane. The length of the axis connecting the four rotating centers of the UAV is *d*, the length of each rotor blade is *L*, and $\varphi_{nm}$ is the initial phase of the *m*-th blade of the *n*-th rotor. The direction of the electromagnetic waves emitted by radar has a certain detection area. When radar detects unmanned aerial vehicles (UAVs), when the UAV flies into the detection area of the radar, the UAV reflects the echo radiated by the radar onto its own surface. After the radar receives the echo from the UAV, the radar detection processing system processes the echo signal.

To detect unmanned aerial vehicles, it is necessary to process the echo data output by the radar. The radar range-Doppler planar graph is one of the core technologies in radar signal processing, mainly used for distance and speed information of targets. Its core principle is to convert the echo signal received by the radar from the time domain to the frequency domain. By analyzing the distribution of the signal at different frequencies, the position and motion state of the UAV can be detected.

### 2.2. The Mathematical Model of the UAV Echo

When the body of the UAV is moving at a speed of $v(t)=v_{0}+at$, its discrete motion coordinates are $\left(x_{k},y_{k},z_{k}\right)$, $v_{0}$ is the initial velocity, and a is the acceleration. Define $R_{n}$ as the distance between the radar and the center of each rotor of the UAV. If *P* is a certain scattering point on the *n*-th rotor blade, the distance from this point to the center of the *n*-th rotor blade is $l_{p}$, then the instantaneous distance from point *P* to the radar can be expressed by Formula (1).

(1) $$R_{p}\left(t\right)=R_{n}+\int_{0}^{t}v(t)dt+l_{p}P\left(t\right),$$

where $P\left(t\right)$ represents the radar transmission power, $P\left(t\right)=\sin\beta \cos(2\pi f_{n}t+\varphi_{nm}+\alpha )$, $f_{n}$ is the rotational frequency of the *n*-th rotor of the UAV, α is the azimuth angle between the radar and the UAV, and v(t) represents the radial velocity of the main body of the UAV.

Define $\left(v_{x}(t),v_{y}(t),v_{z}(t)\right)$ as the radial velocity component of the main body of the UAV, then the radial velocity of the UAV can be expressed by Formula (2).

(2) $$v(t)=v_{x}(t)\cos\alpha +v_{y}(t)\sin\beta ,$$

where β is the pitch angles between the UAV and the radar.

The baseband signal of the scattering point *P* received by the radar is:

(3) $$S_{P_{-gv}}\left(t\right)=\exp\left\{-j4\pi R_{0}/\lambda \right\}\exp\left\{-j4\pi \left(\int_{0}^{t}v(t)dt+l_{P}P\left(t\right)\right)/\lambda \right\},$$

where $R_{0}$ represents the initial distance between the UAV and the radar, and λ is the emission wavelength of the radar [14,15].

The total baseband signal of the *m*-th blade of the *n*-th rotor of the UAV can be acquired from Formula (4).

(4) $$s_{nm\_v}\left(t\right)=L\sin c\left[2\pi LP\left(t\right)/\lambda \right]\exp\left(-j4\pi R_{0}/\lambda \right)\exp\left(-j\Phi_{nm\_v}(t)\right)$$

where $\Phi_{nm\_v}(t)$ is the phase function, L is the length of the rotor blades of the UAV, sinc(⋅) is the Sinker function [16], and sinc(⋅) can be expressed by Formula (5).

(5) $$\sin c\left(x\right)=\left\{\begin{array}{ll} 1, & x=0\\ \frac{\sin\left(x\right)}{x}, & x\neq 0 \end{array}\right.$$

The total baseband echo signal of the UAV can be obtained by Formula (6).

(6) $$f\left(t\right)=\overset{N}{\underset{n=1}{\sum }}\overset{M}{\underset{m=1}{\sum }}\left\{L\sin c\left[2\pi LP\left(t\right)/\lambda \right]\cdot \exp\left[-j4\pi \left[R_{n}+\int_{0}^{t}v(t)dt+0.5Lp\left(t\right)\right]/\lambda \right]\right\}$$

where *N* is the number of rotors of the UAV, and *M* is the number of blades of each rotor.

We take the fuselage of the unmanned aerial vehicle (UAV) as a rigid body. Its translational motion will also generate corresponding echo signals, and its baseband echo signal can be obtained by Formula (7).

(7) $$f_{b}(t)=A\exp\left[-j\frac{4\pi f_{0}}{c}\left(R_{0}+\left(\int_{0}^{t}\left(v_{x}(t)\cos\alpha +v_{z}(t)\sin\beta \right)dt\right)t\right)\right]$$

where *A* is the scattering coefficient of the UAV’s fuselage, $f_{0}$ is the carrier frequency of the radar’s transmitted signal, and c is the emission frequency velocity.

The total echo of the unmanned aerial vehicle (UAV) that the radar obtains can be represented Formula (8).

(8) $$\begin{array}{l} S\left(t\right)=f\left(t\right)+f_{b}\left(t\right)=\overset{N}{\underset{n=1}{\sum }}\overset{M}{\underset{m=1}{\sum }}\left\{L\sin c\left[2\pi LP\left(t\right)/\lambda \right]\cdot \exp\left[-j4\pi \left[R_{n}+\int_{0}^{t}v(t)dt+0.5Lp\left(t\right)\right]/\lambda \right]\right\}\\ \;\;\;+\exp\left[-j\frac{4\pi f_{0}}{c}\left(R_{0}+\left(\int_{0}^{t}\left(v_{x}(t)\cos\alpha +v_{z}(t)\sin\beta \right)dt\right)t\right)\right] \end{array}$$

Based on the established unmanned aerial vehicle (UAV) echo model and taking the received echo model as the foundation, we utilize the principle of generating range-Doppler (RD) planar to convert the received UAV echo signals into range-Dopplermap planar (RD) graphs. When the electromagnetic waves emitted by the radar encounter the UAV target, the reflected echo signal will carry the position and speed information of the UAV target. The distance information is calculated through the time of signal propagation, and the speed information is calculated through the frequency offset caused by the Doppler effect. In signal processing, the raw data received by the radar are a time-domain signal. Through Fast Fourier Transform (FFT), it is converted into a frequency-domain signal, generating a range-Doppler two-dimensional matrix. The horizontal axis represents the distance unit, and the total axis represents the Doppler frequency. The intensity of each point corresponds to the reflected energy of the target. In this paper, based on the radar RD planar graph as the input, we use the improved YOLOv8 network to detect UAV.

## 3. The UAV Detection Method Based on YOLOv8-RFL

### 3.1. Overall Idea of the Detection of UAV

**Figure 2:**
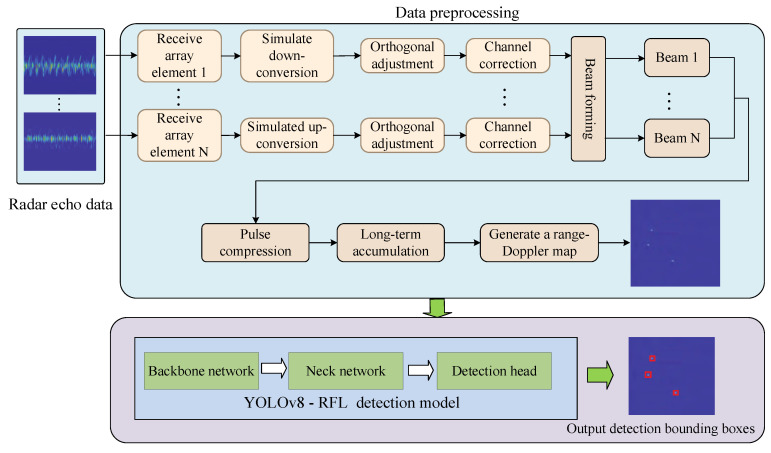
The overall idea of the echo signal detection of UAV.

According to the echo model of UAV based on radar detection technology, it can be seen that the outputs of the radar sensor are time-domain signals, and in order to detect the UAV, the time-domain echo signals the need to be processed. The main idea is: first, collect the echo data from the radar sensor; second, generate the RD planar graph; finally, acquire the UAV target by using the YOLOv8-RFL detection model. Figure 2 shows the overall idea of the echo signal detection of UAV.

In radar signal processing, after generating the range-Doppler planar graph, the range axis in the RD planar graph reflects the range from the target to the radar. The distance resolution can be significantly improved through pulse compression techniques, allowing for the distinction of UAV targets at close distances. The Doppler axis in the RD planar graph reflects the radial velocity of the UAV target. Different velocities of UAV targets can be distinguished by Doppler processing. The key steps from receiving the echoes to forming the dataset are:

(1) Radar signal reception. Each receiving array element unit independently acquires the signal reflected back from the UAV target. In general, to ensure precise signal processing, the data from the receiving array element is externally corrected to eliminate errors introduced by hardware or environmental factors. Through down-conversion and quadrature demodulation, the echo signal of UAV is converted to a baseband signal, which makes subsequent digital signal processing easier.
(2) The signals processed by channel correction down-conversion and orthogonal demodulation, it can maintain the consistency of each receiving channel, and reduce the phase and amplitude errors between channels, and thus guarantee the correct combination of multi-channel signals.
(3) Perform beamforming on the corrected signal. Beamforming is the weighted combination of data from each receiving channel in a specific direction to achieve spatial filtering, thereby generating beams in different directions to enhance the directionality of the UAV target signal while suppressing interference from other directions. Based on this, pulse compression is applied to the received radar echo signals to improve range resolution. The long-time accumulation method is introduced to perform Doppler processing on the pulse-compressed signal to form the RD planar graph.
(4) The range-Doppler map is formed by long-term accumulation process. The long-term accumulation process mainly includes moving target detection (MTD) and moving target indication (MTI). MTD performs Doppler processing on the signal after pulse compression, and MTI further suppresses static or slow background noise to highlight moving targets. Based on the RD planar graph, the YOLOv8-RFL detection model is used to detect the UAV target.

The dataset preparation for this paper is mainly carried out during the experiment. The radar is set up in an open and relatively spacious area with a wide field of view to ensure that the propagation of the radar signal and the reception of the echo minimize environmental interference as much as possible. One to three quadcopters of unmanned aerial vehicles are used as the detection objects, and different motion mode tests are conducted, respectively, including uniform linear motion and non-uniform motion. By collecting data from unmanned aerial vehicle (UAV) targets in different motion states, the detection performance of the radar system under various motion modes of UAV targets can be verified. The setting of this experimental environment aims to eliminate the interference of multipath effects and terrain on radar echo signals, ensuring that the collected data can truly reflect the motion characteristics of the target and providing reliable basic data for subsequent algorithm verification. In addition, we use the LabelImg 2023 software to label the data, highlighting the bright spots in the RD planar graph. A.txt file was generated for each image. After the annotation is completed, the dataset is classified into the training set, validation set and test set. Based on these datasets, we can verify the proposed algorithm.

### 3.2. The UAV Detection Method

#### 3.2.1. The Design of the YOLOv8-RFL Detection Model

**Figure 3:**
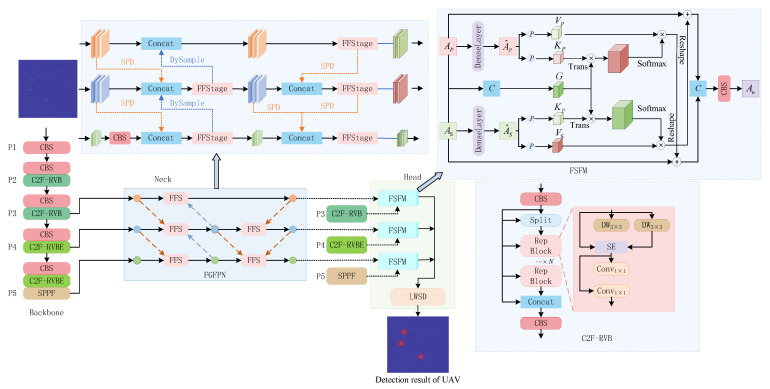
The YOLOv8-RFL detection model.

In this paper, we design the YOLOv8-RFL network detection model, this detection model is mainly based on the YOLOv8 basic network, constructing new neural network modules C2f-RVB and C2f-RVBE, and proposing through the reparameterization convolutions Block (RepBlock) and EMA attention mechanisms have enhanced the feature extraction capability of the backbone network. The focusing generalized feature pyramid networks (FGFPNs) based on feature focusing is proposed, and the feature focus stage (FFStage) is utilized. The FFStage and feature diffusion strategies enhance the feature fusion and interaction among various levels of the network. Construct the feature semantic fusion module (FSFM) based on the cross-attention mechanism to integrate the complementary shallow features of the backbone network and the deep features of the neck network. Design a lightweight weight sharing detection head (LWSD) based on weight sharing, optimize the model scale, and improve the detection efficiency of the UAV by the designed model. Through the improved YOLOv8, we integrated reparameterization convolutions Block (RepBlock), focusing generalized feature pyramid networks (FGFPNs) and a lightweight weight sharing detection head (LWSD) to form a new detection model, the new detection model is called YOLOv8-RFL, Figure 3 is the YOLOv8-RFL model.

The YOLOv8-RFL model proposed in this paper has the following characteristics:

(1) The radar RD planar graph is input from the backbone network, and the UAV target is extracted from the complex background through the C2f-RVB and C2f-RVBE modules to acquire a feature map containing multi-scale UAV feature information. Based on the FGFPN neck network, the model can better aggregate feature maps from different resolutions of the backbone network, improving the efficiency and accuracy of feature fusion.
(2) The shallow features from the backbone network and the deep features from the neck network are integrated through the FSFM to generate high-quality fused feature maps with rich details and deep semantic information. The LWSD detection head is utilized to perform more accurate classification based on the generated fused feature maps and output the detection results of the UAV information.

#### 3.2.2. The Improved C2f Feature Extraction Module

**Figure 4:**
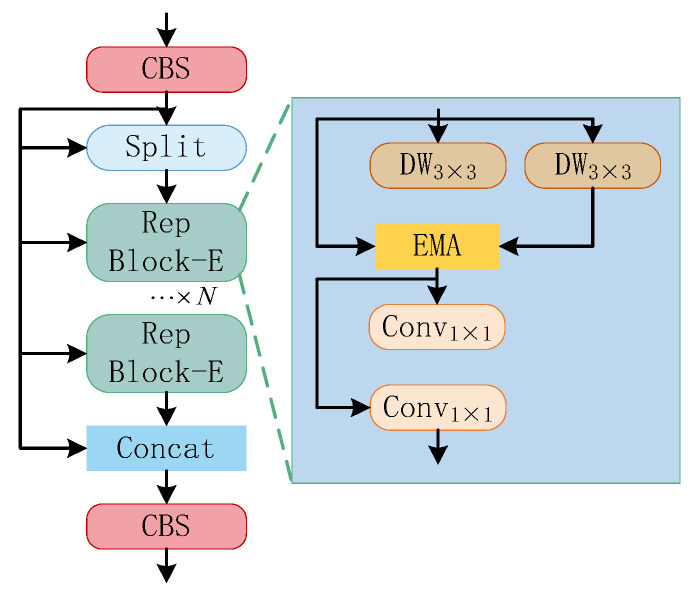
Structure of C2f-RVBE modules.

Because the background, where the UAV is located is often complex, the backbone network is vulnerable to background interference when extracting the UAV echo features. The initial C2f module only uses convolutional layers for feature extraction and transformation [17,18], making it difficult for the model to distinguish between background factors and UAV echo features, thereby affecting the efficiency and accuracy of UAV feature extraction. To enhance the extraction ability of the backbone network for UAV echo features, the C2f module is improved by combing the RepViT algorithm architecture, and the improved C2f feature extraction module is shown in Figure 4.

The RepBlock is the key part of the C2f-RVB module for feature extraction. In the traditional C2f-RVB [19,20,21], the feature map inputted from the network port is extracted by DW Conv for local spatial information. The feature channels are dynamically weighted by the SE layer, and finally the feature is output through a series of convolutional layers for feature blending. RepBlock optimizes computational efficiency through DW Conv, while using SE layers to enhance the ability to extract key feature information. The overall processing flow of the C2f-RVB module can be represented by Formula (9).

(9) $$F_{1}=\mathrm{Conv}(\mathrm{SE}(F_{0},{\mathrm{DW}}_{1\times 1}(F_{0}),{\mathrm{DW}}_{3\times 3}(F_{0}))),$$

where $F_{0}$ and $F_{1}$ represent the input and output features of the module, Conv(⋅) represent the convolutional layer, SE(⋅) represent the dynamic weighting operation of the layer, DW(⋅) represent the SE depth-separable convolutional layer, and the subscript is the size of the convolutional kernel [22].

The SE layer in RepBlock may lose some feature information when dynamically adjusting the channel. To enhance the model’s ability to capture key features of the UAV echo, the improved C2f-RVB module mainly replaces the SE with the EMA mechanism. The EMA contains two parallel subnetworks, one for acquiring global information in the spatial dimension, and the other for capturing local cross-channel interactions. This parallelism enables the model to learn both local and global features of UAV echo features simultaneously, focusing on useful UAV feature information and enhancing the anti-interference ability of feature extraction in the backbone network. Since deep UAV features are more difficult to extract in the backbone network, shallow features can often be extracted more simply and accurately by the model.

#### 3.2.3. The Generalized Feature Pyramid Network Based on Feature Focusing

**Figure 5:**
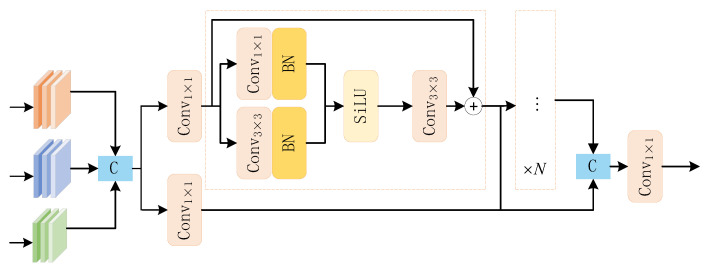
FFStage structure.

The traditional YOLOv8 neck network only uses cascading and up-down sampling operations to fuse the features of different network levels. This relatively simple fusion strategy fails to fully capture the complex relationship between features of different scales, and part of the feature information is not effectively utilized, which results in missed and false detections of UAV echo feature information. To ensure that the model can effectively fuse features of different scales, this paper constructs a new FFStage feature focusing module and proposed the FGFPN neck network to improve the fusion quality of UAV echo features. Unlike the neck network of YOLOv8, the proposed YOLOv8-RFL sets up the FFStage module in the FGFPN neck network, which can accept feature inputs from up to three scales, and contains multiple re-parameterized convolutional modules internally, using parallel convolutional groups to capture rich cross-scale context information, effectively reducing the amount of computational and the model complexity while maintaining the quality of feature fusion. Figure 5 shows the FFStage structure.

The parameterized convolution module in FFStage can be expressed by Formulas (10) and (11).

(10) $$\mathrm{Rep}(\cdot )=\mathrm{SiLU}(\mathrm{BN}({\mathrm{Conv}}_{3\times 3}(F_{x}),{\mathrm{Conv}}_{1\times 1}(F_{x}))),$$

(11) $$F_{1}^{i+1}=F_{0}^{i}⊕{\mathrm{Conv}}_{3\times 3}(\mathrm{Rep}(F_{0}^{i})),$$

where i represents the number of modules, i∈[1,n], i=1 represents the first convolutional module, Rep(⋅) represents the re-parameterization operation, BN(⋅) and SiLU(⋅) represents batch normalization and SiLU activation functions, respectively, and ⊕ represents element-by-element addition [23,24,25]. The FFStage module can be expressed by Formula (12).

(12) $$\mathrm{FFStage}(\cdot )={\mathrm{Conv}}_{1\times 1}(C({\mathrm{Conv}}_{1\times 1}(C(X^{t})),\sum_{t=1}^{n}F_{1}^{i})),$$

where $X^{t}$ represents UAV features from different scales, t∈[1,2,3], and when t=1 is the minimum scale feature layer.

The direction of feature information transmission in the YOLOv8 neck network is “bottom-up” and “top-down”, with less interaction between different layers. The FGFPN neck network uses the idea of “feature focusing—diffusion”, combining defect features from different layers through FFStage, and then using multi-level interlayer interactions to diffuse features with rich contextual information to improve the detection accuracy of UAV echo signals.

Although the diffusion strategy of the FGFPN neck network enhances the interaction of UAV echo signal features, it also introduces additional up-and-down sampling operations, which may lead to the loss of some feature information. To eliminate the effects of the additional up-and-down sampling operations, this paper selects dynamic sampling-based DySample as the upsampling operation of the network, while using space-to-depth convolution for downsampling the network, reducing computational complexity and the number of parameters, and avoiding the loss of important information during fusion. DySample is an ultra-lightweight dynamic upsampler that directly controls the sampling process through point sampling, can better restore the details of RD planar graph images and improve the quality of upsampling. Through the feature focusing module of FFStage and the feature diffusion strategy, the FGFPN necking network enhances the transfer of feature information between different layers, and able to make the features at different scales have detailed contextual information, and effectively improves the overall feature fusion ability of the model.

#### 3.2.4. The Feature Semantic Fusion Module Based on Cross-Attention Mechanism

**Figure 6:**
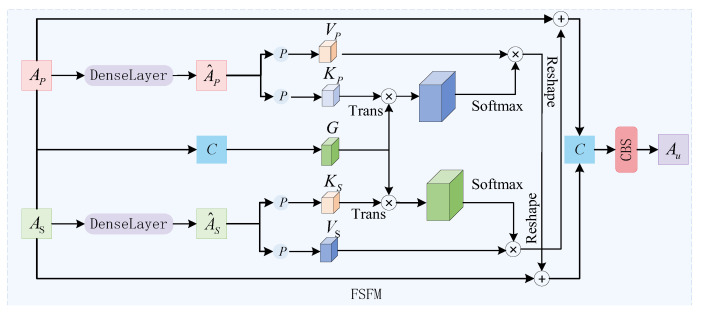
The feature semantic fusion module.

The FGFPN neck network can effectively fuse insulator defect features of different scales efficiently, and the multi-scale semantic information of the deep features output is richer than that of the shallow features of the backbone network. With the deepening of the model feature extraction, the relatively clear shallow feature information in the feature map will gradually become more abstract and fuzzy deep feature information, and the semantic information such as the texture and shape of the UAV features of the radar RD planar graph is fully extracted and fused [26,27]. Because the shallow features typically contain richer detail and structural information, but the semantic information is scarcer, while deep features have richer semantic information but less perception of UAV feature details. To address this issue, this paper proposes a FSFM based on the cross-attention mechanism, which aggregates complementary shallow features of the neck network to meet the semantic requirements of detection head classification and localization tasks. Figure 6 shows the feature semantic fusion module.

The FSFM first enhances the shallow $A_{S}$ and deep features $A_{P}$ of the input using dense layers, and simultaneously integrates the shallow and deep features through concatenation operations as G, and then converts the enhanced features ${\hat{A}}_{S}$ and ${\hat{A}}_{P}$ into key points $K_{x}$ and key values $V_{x}$ respectively, using the projection function that includes convolution and reshaping operations; here, the subscript *x* can represent the subscripts *s* and *p* corresponding to $A_{S}$ and $A_{P}$. The concatenated features G are multiplied element-by-element with the key points $K_{x}$ and key values $V_{x}$ in sequence to acquire features with global context information $A_{x}$. Using the cross-attention mechanism, the global features are added to the original features of another branch, and the resulting features are concatenated along the channel dimension and input into the convolutional layer to acquire the final fused features $F_{\mathrm{fu}}$. The overall processing flow of the feature semantic fusion module can be shown in Equations (13)–(17).

(13) $$K_{x}=\mathrm{Reshape}({\mathrm{Conv}}_{K}^{x}({\hat{F}}_{x})),$$

(14) $$V_{x}=\mathrm{Reshape}({\mathrm{Conv}}_{V}^{x}({\hat{F}}_{x})),$$

(15) $$G=C({\hat{F}}_{s},{\hat{F}}_{p}),$$

(16) $$A_{x}=\mathrm{Softmax}(P⊗K_{x}^{T}),$$

(17) $$A_{u}=\mathrm{Conv}(C(A_{S}⊕\mathrm{Reshape}(A_{P}⊗V_{P}),A_{P}⊕\mathrm{Reshape}(A_{S}⊗V_{S}))),$$

where Reshape(⋅) represents the reshaping operation; C(⋅) represents cascading operations on the channel dimension; ⊗ represents element-by-element multiplication; T represents transposition [28,29,30].

The FSFM can effectively utilize information from different levels to generate high-quality fused feature maps with both rich details and deep semantics, thereby meeting the requirements of UAV information detection tasks in complex scenarios.

#### 3.2.5. The Lightweight Detection Head Based on Weight Sharing

**Figure 7:**
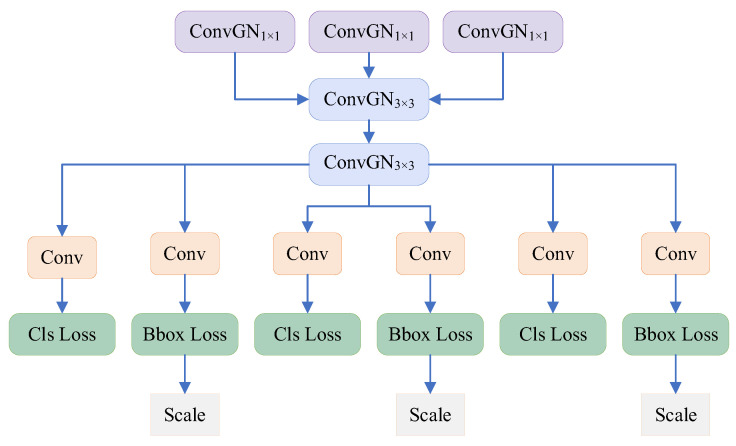
Structure of the lightweight weight sharing detection head.

The detection head section of YOLOv8 uses a decoupled head structure to separate classification and recognition tasks, which allows each branch to focus on classification or recognition tasks and improves overall detection performance, but it also leads to a sudden increase in the number of parameters in the detection head [31]. For this purpose, this paper designs a lightweight detection head based on weight sharing to optimize the model size without sacrificing the performance of the detection head. The structure of the LWSD is shown in Figure 7.

For the output of the neck network, two 3 × 3 shared convolutions in the LWSD apply the same weights to different positions on the insulator feature map, which enables the model to effectively detect UAV information regardless of how its position changes in the radar RD planar graph, improving the generalization ability of the model and helping to reduce overfitting. The convolutional layer in the YOLOv8 detection head uses batch normalization (BN) technique [32,33], which accelerates network training by normalizing the mean and variance of the batch data, but significantly increases model error when the batch size decreases. The LWSD of YOLOv8-RFL proposed in this paper uses Group Normalization (GN) instead of BN, which divides the model channels into multiple groups and calculates the mean and variance separately within each group, which enables the model to maintain relatively stable performance at different batch sizes. Thus, LWSD can significantly reduce the number of parameters required for the detection head, which balances the detection accuracy and operational efficiency of the model, making the model more suitable for the detection of UAV echo signals.

## 4. Experiment and Analysis

### 4.1. Network Training and Radar Parameters

In this experimental environment, the CPU is the Intel-12700F 12-core processor. The GPU is NVIDIA GeForce RTX 3060 Ti and the memory is 32GiB. Development environment: Python 3.9.13, CUDA 12.4, PyTorch 1.13.1. Set the number of epochs for training to 200, batch size to 16, optimizer Adam, initial learning rate to 0.001, sample normalization to 640 × 640. Table 1 shows the experimental configuration information. We adopt the Ka-band linear frequency modulated continuous wave radar system for obtaining the echo signal of the unmanned aerial vehicle (UAV). Table 2 is the main parameter of radar in this study.

To validate the detection model for UAV based on laser detection technology, we conducted experimental analysis using self-built datasets and standard database datasets. In this study, LabelImg 2023 software was used to label the data, the box selected the highlights in the RD planar graph, and a.txt file was generated for each image. After the annotation was completed, the dataset was divided into training set, validation set and test set in an 8:1:1 ratio. Eventually, 3120 images from the training set, 390 images from the validation set and 390 images from the test set were obtained, totaling 3900 images.

### 4.2. Evaluation Indicators

We use the precision (P),the recall rate (R), the mean average precision (mAP, $m_{\mathrm{AP}}$), the Frames Per Second (FPS) and *F*1 to evaluate the performance of the model. Precision (*P*) refers to the proportion of correct predictions among all the results predicted as positive samples. *P* can be defined by Formula (18).

(18) $$P=\frac{T_{P}}{T_{P}+F_{P}}\times 100\%$$

where $T_{P}$ (true positive) indicates that the sample is correctly classified and predicted to be a positive sample; $F_{P}$ (false positive) indicates that the sample is misclassified but is predicted to be a positive sample.

Recall rate (*R*) represents the proportion of the model that successfully detects positive cases among all true positive cases, and it can be defined by Formula (19).

(19) $$R=\frac{T_{P}}{T_{P}+F_{N}}\times 100\%$$

where $F_{N}$ (false negative) indicates that the sample is correctly classified but is predicted to be a negative sample.

$A_{P}$ represents the average precision of each category, it can defined by Formula (20).

(20) $$A_{P}=\int_{0}^{1}P(r)dr$$

Mean average precision (mAP, $m_{\mathrm{AP}}$) represents the average value of all classes of $A_{P}$, and it can be defined by Formula (21).

(21) $$m_{\mathrm{AP}}=\frac{1}{n}\sum_{i=1}^{n}A_{P_{i}}$$

mAP50/% refers to the average detection accuracy of all target categories when the Intersection over Union (IoU) threshold is 0.5. The *F*1 score is a key metric for evaluating the overall performance of the model, taking into account the accuracy of the model’s predictions and its ability to cover positive samples. It can be expressed by Formula (22).

(22) $$F1=\frac{2\times P\times R}{P+R}$$

The FPS metric visualizes the rate at which the model processes radar echo data and outputs detection results, that is, the number of echo signal frames that the model can process per unit of time. A detection model with high FPS can ensure that the system can process and analyze a large number of radar echo signals quickly within a short period of time, enabling real-time monitoring of UAVs.

### 4.3. Experiment Comparative Analysis

#### 4.3.1. Comparative Experiments of Different Models

To verify the effectiveness of the YOLOV8-RFL model on the UAV detection tasks, this paper also selects a relatively typical target detection model and the benchmark model YOLOv8 for experimental comparison, such as Deformable-DETR, YOLOX, Sparse R-CNN, ATSS, and basic YOLOv8. A rather obvious feature of the comparison models we have selected is that all of them have relatively prominent lightweight capabilities. Based on the selected training set, validation set and test set, we conduct training and testing in accordance with the relevant parameters provided in Table 1. Table 3 is the comparison results under the different models.

As can be seen from the performance comparison results in Table 3, YOLOv8-RFL demonstrates outstanding performance in some key indicators. Specifically, the precision (P) reached 87.65%, the recall rate (R) was 84.27%, the mAP50 was as high as 87.14%, and the F1 score was 86.48%, all of which outperformed other comparison models, indicating that the YOLOv8-RFL has obvious advantages in detection accuracy, target coverage ability, and comprehensive performance. At the same time, the FPS of the YOLOv8-RFL was 62.28, which is not only outstanding among all models, but also ensured the model’s efficient real-time processing capabilities in practical applications. This indicates that YOLOv8-RFL not only excellent in detection accuracy but also meets the strict real-time requirements in real scenarios, making it an ideal choice for UAV radar echo signal detection tasks. In addition, both YOLOX and the basic YOLOv8 have relatively good detection accuracy and perform well in the calculation results of other performance indicators.

**Figure 8:**
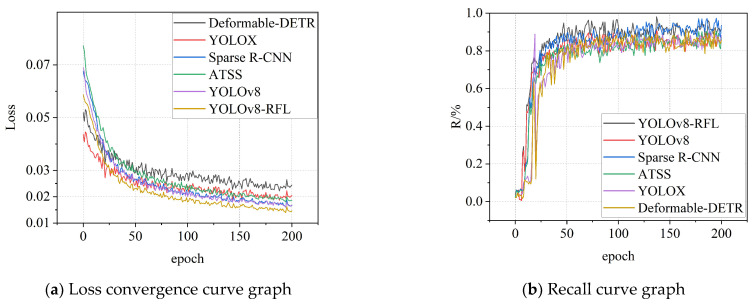
Comparison Experiment Training Results.

Figure 8 shows the loss convergence curves (Figure 8a) and recall curves (Figure 8b) of the six models during the training process. It can be seen from Figure 8a, the loss values of each model gradually decrease as the number of training epochs increases, indicating that the models are constantly learning and optimizing. Among them, YOLOv8-RFL has a faster rate of loss reduction and a lower final loss value, showing good convergence performance. As can be seen from Figure 8b, the recall rates of each model gradually increase with the number of training rounds and eventually stabilize. Among them, YOLOv8-RFL has the higher recall rate, indicating that it has a high recall ability in UAV detection. But, YOLOX, Sparse R-CNN and the basic YOLOv8 also have relatively high recall rates, but they are slightly lower than YOLOv8-RFL in some individual positions; from this result, the proposed algorithm has a clear advantage in complex background.

**Figure 9:**
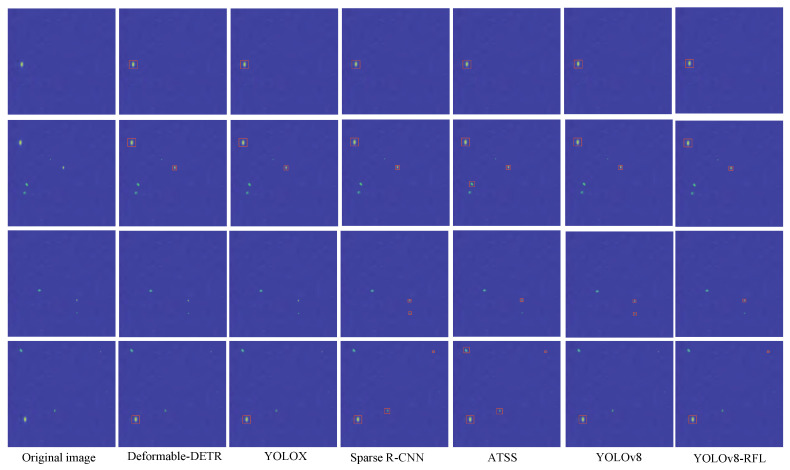
Comparison detection effect under Deformable-DETR, YOLOX, Sparse R-CNN, ATSS, basic YOLOv8 and YOLOv8-RFL.

To further illustrate the rationality of the proposed detection method, based on the test dataset collected by the radar sensor, after RD processing, we combined the YOLOv8-RFL detection algorithm to process the RD planar graph. In this experiment, for the fundamental principles and detection methods of radar detection of unmanned aerial vehicle (UAV) targets, YOLOv8 also effectively detects target as a neural network approach. That is to say, using neural networks for UAV detection on the RD planar map is a modern and effective method, which has many advantages compared with the traditional CFAR method. Figure 9 is comparison detection effect under Deformable-DETR, YOLOX, Sparse R-CNN, ATSS, and basic YOLOv8.

YOLOv8 is the object detection algorithm of the YOLO series. It inherits the forward propagation design of the end-to-end single-stage network and focuses on improving the detection performance of small objects. Neural networks can automatically learn and extract complex features, especially for nonlinear and high-dimensional data. They can capture features that are difficult to capture by traditional methods and integrate multi-level information through a multi-layer network structure. Under the training of big data, neural networks can achieve high-accuracy oral label detection and reduce the false alarm rate. Moreover, neural networks can more intelligently suppress background noise, dynamically adapt to noise and noise in different environments, and improve the robustness of detection. Figure 9 presents the results that reflects the characteristics of various network models. YOLOv8-RFL is mainly aimed at the detection requirements of small targets, it has optimized the traditional anchor-based target area prediction method by adopting an anchor-free strategy, thereby reducing the limitations of anchor box settings on model performance and enhancing the model’s detection sensitivity and generalization ability for small-sized targets. Based on the comparison of different models, it can be seen that there are some differences in the improvement of detection sensitivity and generalization ability for small-sized targets by the models utilized. The main reason is that different detection models have different convolutional layers, pooling layers, attention mechanisms, etc., in their own models, which is also the reason for some differences in detection results just as the comparison results of various models in Figure 9 show, each model has a certain adaptability to the detection environment and to interfering targets.

According to the analysis of the experimental results, it can be known that Deformable-DETR and YOLOX tend to lose relevant information about the defects of small targets of UAVs when the number of network layers increases, resulting in misdetections and missed detections during detection; Sparse R-CNN relies on a fixed number of learnable proposal boxes, and if the initialization is not accurate enough, it will affect the final detection results; ATSS automatically selects positive and negative cases based on the statistical characteristics of the objects, and it is easily interfered by complex environmental factors. In our experiment, we actually collected the echo signals of the UAV. However, due to some interference in the collection environment, the RD planar graph also displayed the information of the interfering targets, the information shown in Figure 9 does indeed include that of UAV and stray targets. Because the unmanned aerial vehicle (UAV) was in motion when obtaining the UAV signal, while other interference sources did not change significantly in the RD map, the characteristics of the UAV could also be observed through the echo function Equations (6)–(8), which contain certain fundamental wave information. From the perspective of the scenarios we tested, the fundamental wave information of the UAV is not very prominent. Through this difference, the detection effect can also be seen in the training results of YOLOv8n-RFL.

The YOLOv8-RFL model can accurately detect UAV, indicating that the proposed model has better detection accuracy in complex backgrounds than other models. Of course, because it is difficult to avoid the presence of non-unmanned aerial vehicle (UAV) targets in the experimental environment, the proposed algorithm also has certain limitations for approximate UAV target information. To further eliminate the interfering targets, we plan to integrate more radars of different dimensions into the current inspection method. Through the echo information of multiple radars, searching for the actual echo correlation characteristic parameters of unmanned aerial vehicles can further enhance the detection capability of the system. In Figure 9, the experiment result can verify the rationality of the proposed detection method and lay the foundation for the next step of research.

#### 4.3.2. The Proposed Method Compared with CFAR

The performance of traditional radar systems in small target detection is limited, especially in complex clutter backgrounds and dynamic target scenarios, where it is difficult to balance high resolution and wide coverage. Traditional radar target detection uses the constant false alarm rate (CFAR) algorithm. CFAR uses a group of Independent and Identically Distributed (IID) reference units adjacent to the detection unit to estimate the clutter power level, and adaptively adjusts the detection threshold as the detection background changes, thereby maintaining a constant false alarm rate so as to effectively detect targets under different environmental conditions. At present, the CFAR algorithm has become quite mature in the field of target detection. However, it is prone to false alarms in noisy environments, especially in scenarios where the noise is intense or changes significantly. A high false alarm rate can affect the overall detection performance. For the fundamental principles and detection methods of radar detection of unmanned aerial vehicle (UAV) targets, YOLOv8 also effectively detects target as a neural network approach. That is to say, using neural networks for UAV detection on the RD planar map is a modern and effective method, which has many advantages compared with the traditional CFAR method. Therefore, in this paper, we mainly use the traditional CFAR method and YOLOv8-RFL detection model for comparison.

About the detection rate, we detected 820 targets in the test set. If the Intersection over Union (IoU) of the predicted bounding box and the true real bounding box is greater than 0.7, it is considered that the target has been detected correctly. The OS-CFAR algorithm for UAV detection is adopted. Table 4 shows the parameters of the OS-CFAR algorithm.

A total of 671 UAV targets were correctly detected by the CFAR method, with a detection rate of 81.92%, while 718 UAV targets were correctly detected by the improved YOLOv8 model, with a detection rate of 87.56%, demonstrating slightly higher detection performance. CFAR is a detection method based on local background, and it assumes that the clutter and the target are independent or have a specific distribution (such as Gaussian distribution or K-distribution). The performance of CFAR will decline under non-uniform background.

YOLOv8-RFL detection model can extract more complex features from input data, capture nonlinear relationships, and utilize the global information of input images to learn the overall characteristics of the UAV target and background. Through large-scale data training, they can automatically adapt to changes in various target characteristics and background noise. In addition, for small targets, the detection performance of CFAR is significantly affected by the size of the window. Especially when the protection unit and reference unit are not adjustable, small targets may be overwhelmed by background interference, resulting in missed detections. Our model has been improved for small target detection, enhancing the detection capability for targets with smaller size and weaker signals, thereby further increasing the detection rate.

#### 4.3.3. Comparative Experiments with the C2f Module

**Figure 10:**
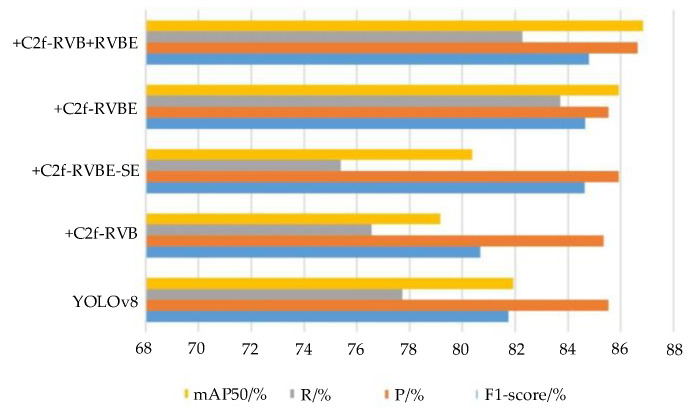
Comparative experiment diagram of C2f modules.

To verify the effectiveness of the C2f module improvements, we conducted comparisons through experiments, and the detailed experimental data are presented in Figure 10.

From the comparison experiments, it can be seen that compared with the initial YOLOv8 model, the improved model using C2f-RVB has improved in detection metrics such as recall rate and mean average accuracy. When the C2f-RVB module was combined with various mainstream attention mechanisms, it was observed that the most significant improvement in model recognition was achieved when combined with the EMA attention mechanism, with the best results in the mAP metric, and the performance of P and R was also good. This indicates that the introduction of the EMA attention mechanism can enhance the model’s ability to capture significant detail information of feature maps in complex backgrounds. By comparison, compared with replacing the backbone network with all C2f-RVB modules or all C2f-RVBE modules, combining C2f-RVB modules and C2f-RVBE modules can achieve a better balance between model detection accuracy and model parameters.

As can be seen from Figure 4, the C2f-RVB and C2f-RVBE modules have better feature extraction capabilities, enabling the model to more accurately detect foreground regions and effectively reducing the interference of complex backgrounds for feature extraction. For UAV targets, the feature boundaries extracted by the C2f-RVBE module are clearer, indicating that the introduction of the EMA attention mechanism can enhance the model’s ability to distinguish between UAV targets and background factors, thereby capturing richer contextual information and enhancing the model’s perception of key feature information.

#### 4.3.4. Ablation Tests

To further validate the effectiveness of the various modules introduced in this paper, the ablation tests were conducted on the UAV echo signal dataset under the same experimental conditions. The basic network framework of YOLOv8 was used as the baseline model, and the improved modules proposed in this paper were added one by one. The ablation results are shown in Table 5. The “√” means that the module is introduced into the network.

The results of the ablation test show that each module has a significant effect on the performance of the YOLOv8 model. The base model shows balanced performance in terms of precision, recall rate, mAP and FPS. With the introduction of the C2f-RVB module, the precision declined slightly but the recall improved. With the addition of the C2f-RVBE module, the precision improved, the recall slightly decreased, and the mAP was further enhanced. The addition of the FFStage module further enhanced precision and recall, and also improved mAP. The introduction of the FSFM continued to improve precision and recall, and the mAP reached 83.54%. The addition of the FGFPN module further enhanced precision and recall, with mAP reaching 84.32%. Ultimately, when all modules were added simultaneously, the model’s precision, recall rate, and mAP all reached their peak at 87.65%, 84.27%, and 87.14%, respectively, while the FPS also increases to 62.28. This indicates that the synergy of the modules significantly enhanced the model’s detection performance, which leads to its optimal performance in terms of precision, recall rate, mAP, and real-time performance.

## 5. Conclusions

In this paper, we research the improved YOLOv8 and form the YOLOv8n-RFL model to detect the echo information of the UAV target based on radar detection technology, and through quantitative experimental processing, the following conclusions were drawn:

(1) In the process of detecting unmanned aerial vehicle (UAV) targets using radar means, because the echo signal formed by UAV belongs to an uncertain state, the signal output by radar sensor appears obvious aliasing, and it is difficult to analyze the target information of UAV quickly and effectively. This paper proposes that the YOLOv8n-RFL detection model is mainly based on the mathematical model of the radar echo signal to form the RD planar graph for identification. By detecting the RD planar graph, it was found that the fusion of the C2f-RVB and C2f-RVBE modules utilized by YOLOv8n-RFL effectively mitigated the impact of complex background information on the extraction of UAV echo information, enabling the backbone network of the model to capture more abundant UAV information and significantly enhancing the model’s ability to extract UAV features.
(2) The proposed FGFPN neck network based on feature focusing adopts the idea of “feature focusing—diffusion”, enhancing the fusion and interaction of features of different scales among various levels of the neck network and improving the expression ability of unmanned aerial vehicle features. In addition, the FSFM based on the cross-attention mechanism is introduced to fuse shallow features with rich detail information and deep features with rich semantic information, avoiding the neglect of key features of UAVs and thereby improving the detection and recognition rate of UAVs.

The YOLOv8n-RFL detection method proposed in this paper focuses on the two-dimensional information of the UAV echo features and can effectively extract the feature information of the UAV from the radar echo signal under complex background and noise interference, achieving high-precision detection of the UAV. However, the method mainly relies on two-dimensional information and has limited ability to acquire precise position and attitude information of the UAV in three-dimensional space. In addition, the robustness of the model in the face of extreme weather conditions or more complex electromagnetic interference needs to be further improved. In the future, it is planned to further optimize the model structure, reduce computational resource consumption, and improve the real-time performance and adaptability of the model to better meet the diverse demands in the practical application of UAV detection.

## Figures and Tables

**Table 1:** Experimental configuration information.

Parameter Name	Parameter Setting
Operating system	Windows
CPU	12th Gen Intel(R) Core(TM) i7-12700F
GPU	NVTDIA CaFnroe RTX 3060 Ti
Python	3.9.13
PyToroh	1.13.1
CUDA	12.4
Training cycle	200
Initial learning rate	0.001
Optimizer	Adam
Batch size	16
Sample normalization	640 × 640

**Table 2:** The main parameter of radar.

Parameter Name	Parameter Value
Working frequency	35.64 GHz
Transmission power	10W (5% duty cycle)
Pulse width	0.1~20 μs
Pulse repetition frequency	32 KHz
Fast sampling frequency	500 MHz
Doppler closure ratio	0.0025
Accumulated frame count	1024
Number of sampling points	1000

**Table 3:** The comparison results under the different models.

Algorithm	P/%	R/%	mAP50/%	F1-Score/%	FPS/ms
Deformable-DETR	81.49	71.27	76.52	75.91	42.62
YOLOX	85.83	77.56	81.17	81.54	57.38
Sparse R-CNN	81.31	75.39	80.38	78.26	42.65
ATSS	81.28	78.31	81.28	79.75	47.65
YOLOv8	85.53	77.72	81.92	81.74	58.24
YOLOv8-RFL	87.65	84.27	87.14	86.48	62.28

**Table 4:** The parameters of the OS-CFAR algorithm.

Parameter Name	Parameter Value
Number of protection units	8
Reference unit number	16
Number of sorting orders	18
Threshold factor	25
Doppler threshold	8
Distance threshold	3
Match speed threshold	7
Match distance threshold	7

**Table 5:** Comparison Results of YOLOv8-RFL ablation tests.

C2f-RVB	C2f-RVBE	FFStage	FSFM	FGFPN	P/%	R/%	mAP/%	FPS
					85.53	77.72	81.92	58.24
√					85.34	80.12	79.17	57.38
	√				85.65	76.56	83.23	57.54
		√			86.30	81.45	84.76	58.57
			√		85.75	79.81	83.54	58.19
				√	86.00	80.50	84.32	58.31
√	√	√	√	√	87.65	84.27	87.14	62.28

## Data Availability

The data that support the findings of this study are available from the corresponding author upon reasonable request.
